# Supplementary figures and images for: The Flexiscope: a low cost, flexible, convertible and modular microscope with automated scanning and micromanipulation
Source: R Soc Open Sci. 2020 Mar 4;7(3):191949. doi: 10.1098/rsos.191949 (PMC7137931; doi:10.1098/rsos.191949)

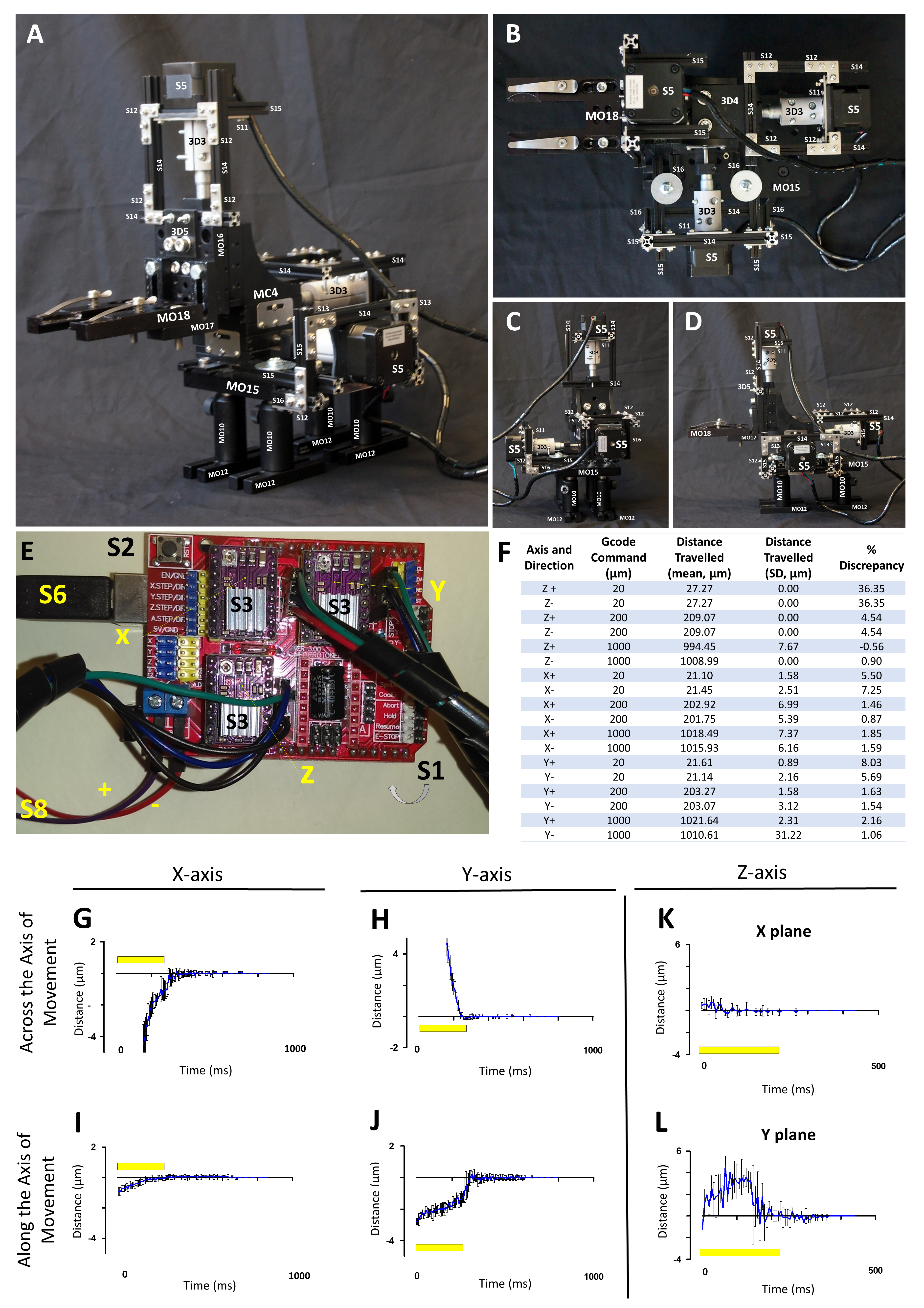

Supplement: Figure S1 [file rsos191949supp1.tif]

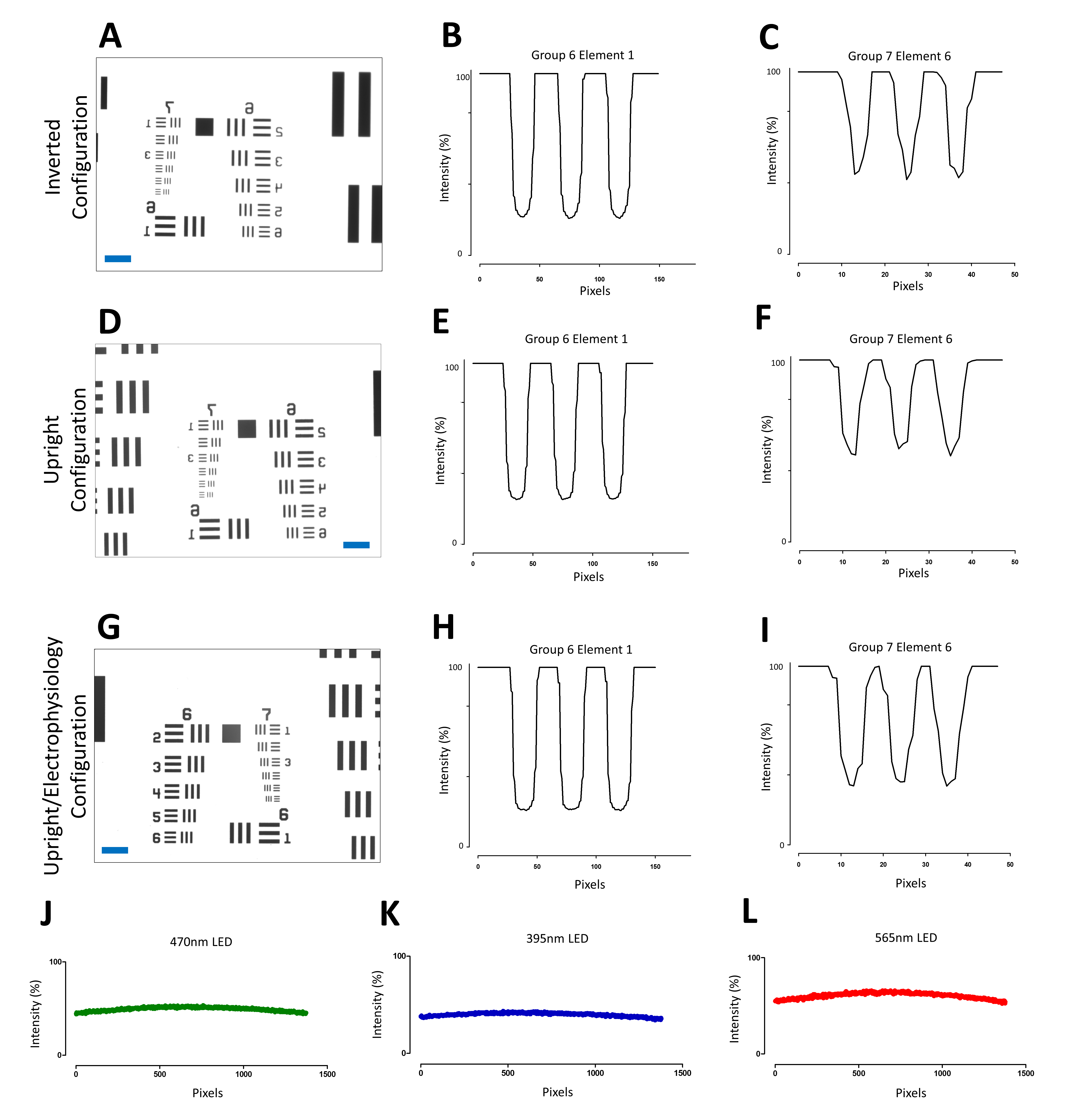

Supplement: Figure S2 [file rsos191949supp2.tif]

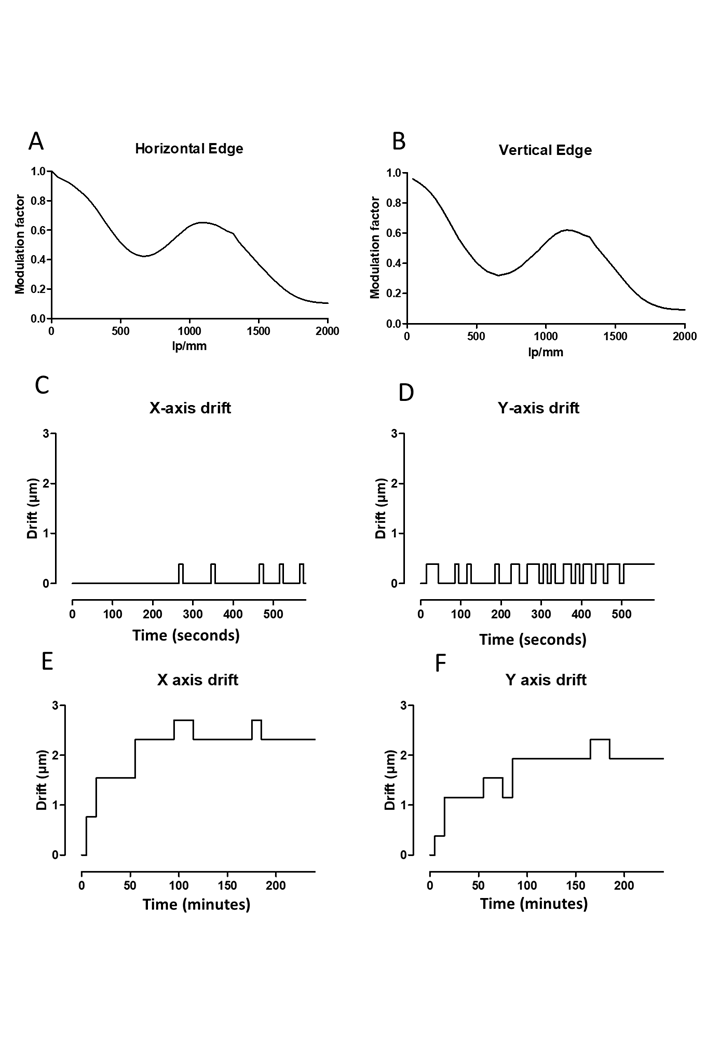

Supplement: Figure S3 [file rsos191949supp3.tif]
