# Supplementary material for: The Flexiscope: a low cost, flexible, convertible and modular microscope with automated scanning and micromanipulation
Source: R Soc Open Sci. 2020 Mar 4;7(3):191949. doi: 10.1098/rsos.191949 (PMC7137931; doi:10.1098/rsos.191949)

User Guidelines: Upright Flexiscope Assembly

The Flexiscope: a Low Cost, Flexible, Convertible, and Modular Microscope with Automated Scanning and Micromanipulation.

Amy Courtney*, Luke Alvey, George O.T. Merces, and Mark Pickering.
School of Medicine, University College Dublin, Ireland.
*Correspondence: amy.courtney@ucdconnect.ie

October 2019

This instruction manual describes how to assemble the Flexiscope in the upright configuration.

As described in section 2.1 of the manuscript, the core optical components can be divided into three functional units: the fluorescence illumination module, the infinite space module and the camera module. Here, we will describe how to assemble each module and then combine and mount them on an optical table for an upright configuration. This systems utilises threaded 1" diameter lens tubes.

Each individual component has been allocated a part designation, for use throughout this instruction manual and within the manuscript. Table 1 lists each part and its designation.

1. The Fluorescence Illumination Module

1.1. Begin by fitting each dichroic mounting cube (C07) with their designated dichroics (CO8 and CO9). The dichroics are mounted in the cubes as described on the manufacturer's website.

1.2. The dichroics are incorporated into CO7 at 45° angles. There is a line mark on the side with the dichroic coating. The LED illumination from CO2 and CO3 should be directed towards this side of the respective filters (CO8 and CO9). See Figure 1.

1.3. Seal the open holes in the cubes with dust caps (CO38).

1.4. Combine the cubes (CO7) using a coupler (CO27). See Figure 2 B.

1.5. Each LED (CO1, CO2 & CO3) is collimated using an aspheric condenser lens (CO6). The lens is placed in CO37 and held in place with a retaining ring using an adjustable spanner wrench (T1).

1.6. The distance from the LED to the lens should be 20mm and was measured with an electronic calibres (Figure 3). Further details are available on the manufacturer's website. LED collimation testing is explained in section 5 of this document.

1.7. Each collimated LED is attached to the dichroic mounting cubes (CO7) using a coupler (CO27).

1.8. The Iris (CO10) is incorporated into the system using CO33 and CO27 (Figure 2 B). Note: this component is optional. We found that the iris caused issues with uneven illumination and it was therefore omitted in later configurations of the Flexiscope.

2. The Infinite Space Module

2.1. Begin by configuring the swappable filter set mounts (CO15). The three CO15s contain an excitation, emission and dichroic filter corresponding to either 630nm (Texas Red, CO12), 530nm (FITC, CO13) or 460nm (BFP, CO14) emission wavelengths. The filters are incorporated into the mount as described on the manufacturer's website.

2.2. The main component in the infinite space module is the filter mount-holding cube (CO16). It is connected to the fluorescence illumination module via the iris/CO27 as seen in Figure 2 B.

2.3. Attach the turning mirror (CO11) to the filter mount-holding cube with the cage cube connector (CO29) as seen in Figure 2 D.

2.4. Attach the lens turret (CO18) to the filter mount-holding cube with CO34 and attach the infinite conjugate objectives (CO18, CO19) to the lens turret as seen in Figure 2 D.

2.5. Place the achromatic doublet lens in CO36 with a retaining ring and adjustable spanner wrench (T1).

2.6. The turning mirror (CO11) is attached to the achromatic doublet lens (CO21) via CO27 as seen in Figure 2 A.

3. The Camera Module

3.1. The distance between the camera sensor and the achromatic doublet lens (CO21) should be 150mm. This is achieved by combining CO31, CO28, CO35, and CO32.

3.2. Ensure that CO31 and CO32 has the M4 mount point orientated downwards (Note: reconfiguring to the inverted configuration means the point at which the achromatic doublet is couplet to the mirror will need to be rotated to enable mounting. Do not change the distance after the achromatic to the camera sensor during reconfiguration).

3.3. Four alignment posts (CO30) are also attached as seen in Figure 2 A & C.

3.4. The camera is then attached to CO32 with a C-mount adapter (CO24). We used a 3D printed component to align the camera (3D4).

4. Mounting and Motion Control Components

4.1. Two optical posts (MO4) are attached to the dichroic mounting cubes (CO7) and combined with post holders (MO11) and post mounts (MO13). See Figure 2 D.

4.2. An optical post (MO5) is attached to the cage plate (CO32). A post holder (MO11) and post mount (MO13) is also used as seen in Figure 2 A.

4.3. The entire scope is then attached to the lab jack (MC1) via the post mounts using M6 bolts as seen in Figure 2 D.

4.4. The piezoelectric actuators (MC2) are incorporated into the XYZ translational stage (MC4) as seen in Figure 2 B.

4.5. Specimen holder configuration on XYZ stage: base plate (MO16), two right angle brackets (MO17) and a microscopy slide holder (MO18). See Figure 2 D.

4.6. The XYZ stage is mounted to a small breadboard (MO15).

4.7. Optical posts (MO10), post holders (MO8) and post mounts (MO13 or MO12) mounted on the breadboard/stage.

4.8. The breadboard/stage and the scope/lab jack can be aligned to each other and then mounted directly to the main breadboard (MO19) using M6 bolts.

5. Calibration

5.1. Testing LED collimation:

- In order to achieve diffuse flat illumination on your sample LED collimation may need to be adjusted. Using the fluorescent slides (CA2) show a live preview of the camera on the point grey FlyCapture software. A live histogram can be opened in the software. Adjust CO37 until the histogram is flat as seen in Figure 4.

- For the 565nm LED use the red slide, for the 470nm LED use the green slide and for the 395nm LED use the blue slide.

5.2. Determining pixel size:

- Determining pixel size: Acquire an image of a calibration slide and measure length of defined distance and convert from pixels to µm using imageJ as seen in Figure 5.

Table 1. Flexiscope Component List with Designators and a Cost Description.

Component	Part Designator	Component Description	Product Code	Supplier	Quantity	Cost per unit (€)	Total cost (€)
Core Optical Components							
**Fluorescence Illumination Module**	CO1	Lime (565 nm) Mounted LED	M565L3	ThorLabs	1	186.10	186.10
	CO2	Blue (470 nm) Mounted LED	M470L3	ThorLabs	1	236.38	236.38
	CO3	UV (395 nm) Mounted LED	M395L4	ThorLabs	1	249.61	249.61
	CO4	LED Driver	LEDD1B	ThorLabs	3	258.43	775.29
	CO5	Power Supply for LED and Piezo T-Cubes	TPS008	ThorLabs	1	158.76	158.76
	CO6	Aspheric Condenser Lens (for LED collimation)	ACL2520U-DG6-A	ThorLabs	3	24.43	73.29
	CO7	Dichroic mounting cube	CM1-DCH/M	ThorLabs	2	139.36	278.72
	CO8	GFP Dichroic Filter (Refl. Band = 452-490 nm, Trans. Band = 505-800 nm)	MD498	ThorLabs	1	188.87	188.87
	CO9	BFP Dichroic Filter (Refl. Band = 360-407 nm, Trans. Band = 425-575 nm)	MD416	ThorLabs	1	188.87	188.87
	CO10	Iris Diaphragm	SM1D12D	ThorLabs	1	56.71	56.71
**Infinite Space Module**	CO11	Protected Silver Tuning Mirror	CCM1-P01/M	ThorLabs	1	144.90	144.90
	CO12	Texas Red Filter Set (Excitation, Emission, and Dichroic)	MDF-TXRED	ThorLabs	1	551.25	551.25
	CO13	FITC Filter Set (Excitation, Emission, and Dichroic)	MDF-FITC	ThorLabs	1	551.25	551.25
	CO14	BFP Texas Red Filter Set (Excitation, Emission, and Dichroic)	MDF-BFP	ThorLabs	1	551.25	551.25
	CO15	Swappable Filter Set Mount	DFMT1	ThorLabs	3	177.28	531.84
	CO16	Filter Mount Holding Cube	DFMB/M	ThorLabs	1	90.85	90.85
	CO17	Filter Cube Blank Top Plate	DFM1C	ThorLabs	1	32.40	32.40
	CO18	Objective Lens Turret	OT1	ThorLabs	1	262.53	262.53
	CO19	4X Olympus Plan Achromat Objective (NA=0.10, WD=18.5 mm)	RMS4X	ThorLabs	1	167.07	167.07
	CO20	20X Olympus Water Immersion Objective (NA=0.5, WD=3.5 mm)	UMPLFLN20XW	Masons	1	1,176.68	1,176.68
**Camera Module**	CO21	Tube lens: Achromatic Doublet (f=150.0 mm)	AC254-150-A	ThorLabs	1	62.56	62.56
	CO22	USB 3.0 Monochrome Camera (Flea®3 ½" FL3-U3-13Y3M-C)	86-767	Edmund Optics	1	783.75	783.75
	CO23	USB 3.0 Color Camera (Grasshopper GS3-U3-15S5C-C 2/3")	33-533	Edmund Optics	1	1,435.09	1,435.09
	CO24	1" Lens Tube to C-Mount Adapter (Male to Male)	SM1A39	ThorLabs	1	18.60	18.60
	CO25	USB 3.0 Locking Cable	86-770	Edmund Optics	1	23.75	23.75
	CO26	Infrared LED array	N/A	Salvaged from CCTV Camera	1	N/A	N/A
**Coupling and Alignment**	CO27	Male to Male Tube Coupler (0.5")	SM1T2	ThorLabs	6	17.55	105.30
	CO28	Male to Male Tube Coupler (2")	SM1T20	ThorLabs	1	18.74	18.74
	CO29	Cage Cube Connector	C4W-CC	ThorLabs	1	43.57	43.57
	CO30	Alignment Posts (Diameter = 6 mm, L = 75 mm)	MS3R/M	ThorLabs	4	7.10	28.40
	CO31	1" Threaded Cage Plate (0.50" Thick)	CP02T/M	ThorLabs	1	17.90	17.90
	CO32	1" Threaded Cage Plate (0.35" Thick)	CP02/M	ThorLabs	2	14.11	28.22
	CO33	1" Lens Tube, 0.30" Thread Depth	SM1L03	ThorLabs	4	10.72	42.88
	CO34	1" Lens Tube, 0.5" Thread Depth	SM1L05	ThorLabs	3	11.33	33.99
	CO35	1" Lens Tube, 3" Thread Depth	SM1L30	ThorLabs	1	21.95	21.95
	CO36	1" Lens Tube, 0.31" Travel Range (Adjustable)	SM1V05	ThorLabs	2	26.11	52.22
	CO37	1" Lens Tube, 0.81" Travel Range (Adjustable)	SM1V10	ThorLabs	1	29.34	29.34
	CO38	Plastic Dust Cap for 1" Lens Tubes	SM1EC2	ThorLabs	10	1.76	17.64
	CO39	1" Lens Tube, 1.5" Thread Depth	SM1L15	ThorLabs	1	14.27	14.27
Other components							
**Motion Control Components**	MC1	Lab Jack	L490/M	ThorLabs	1	527.40	527.40
	MC2	Piezoelectric Actuator (13 mm Travel)	PIA13	ThorLabs	4	440.12	1,760.48
	MC3	T-Cube: Piezoelectric Actuator Controller	TIM101	ThorLabs	1	837.90	837.90
	MC4	13 mm XYZ Translation Stage	MT3/M	ThorLabs	1	798.21	798.21
	MC5	13 mm Translation Stage Plate in One Dimension	MT1A/M	ThorLabs	1	356.00	356.00
	MC6	25 mm XYZ Translation Stage	PT3A/M	ThorLabs	1	954.00	954.00
**Mounting Components**	MO1	Optical Post (Ø12.7 mm, L = 30 mm)	TR30/M-P5	ThorLabs	5	3.84	19.20
	MO2	Optical Post (Ø12.7 mm, L = 20 mm)	TR20/M-P5	ThorLabs	5	3.76	18.82
	MO3	Optical Post (Ø12.7 mm, L = 50 mm)	TR50/M-P5	ThorLabs	5	4.12	20.60
	MO4	Optical Post (Ø12.7 mm, L = 75 mm)	TR75/M-P5	ThorLabs	5	4.39	21.95
	MO5	Optical Post (Ø12.7 mm, L = 100 mm)	TR100/M-P5	ThorLabs	5	4.50	22.52
	MO6	Optical Construction Post for TR75C/M (Ø12.7 mm)	TR75T/M	ThorLabs	1	14.90	14.90
	MO7	Optical Construction Post- Variable Angle (Ø12.7 mm)	TR75C/M	ThorLabs	1	14.90	14.90
	MO8	Post Holders (Ø12.7 mm, L=30 mm)	PH30/M-P5	ThorLabs	5	6.33	31.64
	MO9	Post Holders (Ø12.7 mm, L=20 mm)	PH20/M-P5	ThorLabs	5	6.20	31.01
	MO10	Post Holders (Ø12.7 mm, L=50 mm)	PH50/M-P5	ThorLabs	5	6.79	33.96
	MO11	Post Holder (Ø12.7 mm, L=75 mm)	PH75/M-P5	ThorLabs	5	7.05	35.25
	MO12	Mounting Base (25 mm x 75 mm x 10 mm)	BA1/M-P5	ThorLabs	5	4.45	22.23
	MO13	Mounting Base (25 mm x 58 mm x 10 mm)	BA1S/M-P5	ThorLabs	5	3.99	19.94
	MO14	Mounting Base (50 mm x 75 mm x 10 mm)	BA2/M-P5	ThorLabs	5	5.80	28.98
	MO15	Breadbord (100 mm x 150 mm x 12.7 mm)	MB1015/M	ThorLabs	1	36.90	36.90
	MO16	Base Plate for Z-Axis Stage Mounting (65 mm x 65 mm x 10 mm)	UBP2/M	ThorLabs	1	30.87	30.87
	MO17	Right-Angle Bracket	AB90C/M	ThorLabs	2	22.67	45.34
	MO18	Microscopy Slide Holder	MAX3SLH	ThorLabs	1	105.69	105.69
	MO19	Composite Core Optical Breadboard (600 x 600 x 28 mm)	M-TD-22	Newport	1	488.70	488.70
	MO20	Sorbothane Feet (Ø27.0 mm)	AV4/M	ThorLabs	4	4.69	18.74
**Blackout Enclosure**	BE1	Black Hardboard (610 mm x 610 mm, 5 mm Thick)	TB4	ThorLabs	3	18.81	56.43
	BE2	Aluminium Extrusion (25 x 25mm, L = 600 mm)	XE25L600/M	ThorLabs	8	25.52	204.16
	BE3	Vertical Blackout Blind (600 mm x 1700 mm)	VBB060	ThorLabs	2	88.11	176.22
	BE4	Black Masking Tape (50mm x 55m)	T137-2.0	ThorLabs	1	13.86	13.86
**Head Stage Mounting**	HS1	Male M6 to Female M4 Coupler	AS4M6M	Thorlabs	2	3.81	7.62
	HS2	Male M4 to Female M3 Coupler	MSA4/M	Thorlabs	2	4.17	8.34
	HS3	45° Brackets	100281	MakerBeam	12	0.58	6.95
	HS4	Aluminum Extrusion (10x10mm, L= 100mm)	100078	MakerBeam	16	0.58	12.25
Additional configuration parts							
**3D printed Components**	3D1	Swappable Filter Set Mount Holder			3	N/A	N/A
	3D2	Lens Tube - Part1			1	N/A	N/A
	3D3	Lens Tube - Part2			1	N/A	N/A
	3D4	Camera Alignment	*stl files available in supplementary file 5*		1	N/A	N/A
	3D5	Coupler: Actuator to Stepper			3	N/A	N/A
	3D6	Y-axis Mounting			1	N/A	N/A
	3D7	Z-axis Mounting			1	N/A	N/A
**Stepper Motor XYZ-Stage**	S1	Arduino Uno	A000066	Farnell	1	17.15	17.15
	S2	CNC Shield V3.0	N/A	gearbest.com	1	4.28	4.28
	S3	DRV8825 Motor Drivers	RB-Pol-272	RobotShop	5	1.44	7.19
	S4	Jumpers	791-6454	Radionics	20	0.23	4.62
	S5	NEMA17 Stepper Motor 1.8°, 0.22nm, 2.8 V, 1.33 A, 4 Wire	535-0467	Radionics	3	24.09	72.27
	S6	Male USB A to Male USB B	529-8274	Radionics	1	6.21	6.21
	S7	Female Shorting Link	251-8682	Radionics	10	0.03	0.27
	S8	12V Power Supply	148-963	Radionics	1	12.89	12.89
	S9	Spiral Binding	446-172	Radionics	1	7.21	7.21
	S10	Bearings	100438	MakerBeam	10	1.40	14.00
	S11	NEMA17 Stepper Bracket	100225	MakerBeam	3	3.50	10.50
	S12	90° Brackets	100304	MakerBeam	12	0.58	6.95
	S13	Right Angle Brackets	100326	MakerBeam	12	0.58	6.95
	S14	100mm Anodised MakerBeam	100078	MakerBeam	16	0.77	12.25
	S15	60mm Anodised MakerBeam	100056	MakerBeam	8	0.38	3.00
	S16	40mm Anodised MakerBeam	100056	MakerBeam	8	0.38	3.00
**Calibration**	CA1	1951 USAF Resolution Test Target	R3L1S4P	ThorLabs	1	194.82	194.82
	CA2	Fluorescent Calibration Microscope Slides	FSK5	ThorLabs	1	16.85	16.85
**Tools**	T1	Adjustable Spanner Wrench	SPW801	ThorLabs	1	91.75	91.75

Figure 1. Schematic representation of Flexiscope with a clear view of the fluorescent illumination module.

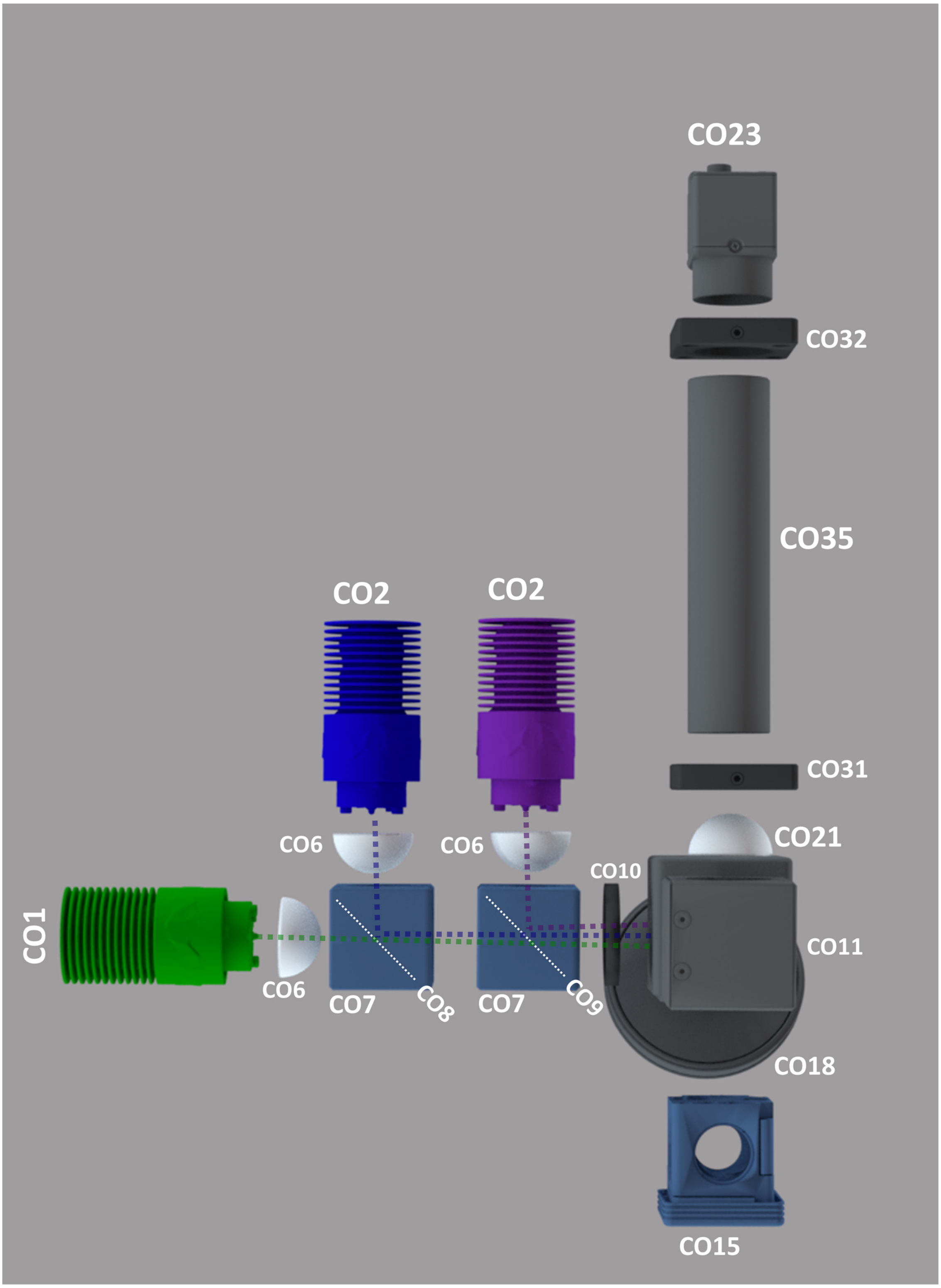

Figure 2. Photographs of the Flexiscope in the upright configuration with part designators labelled

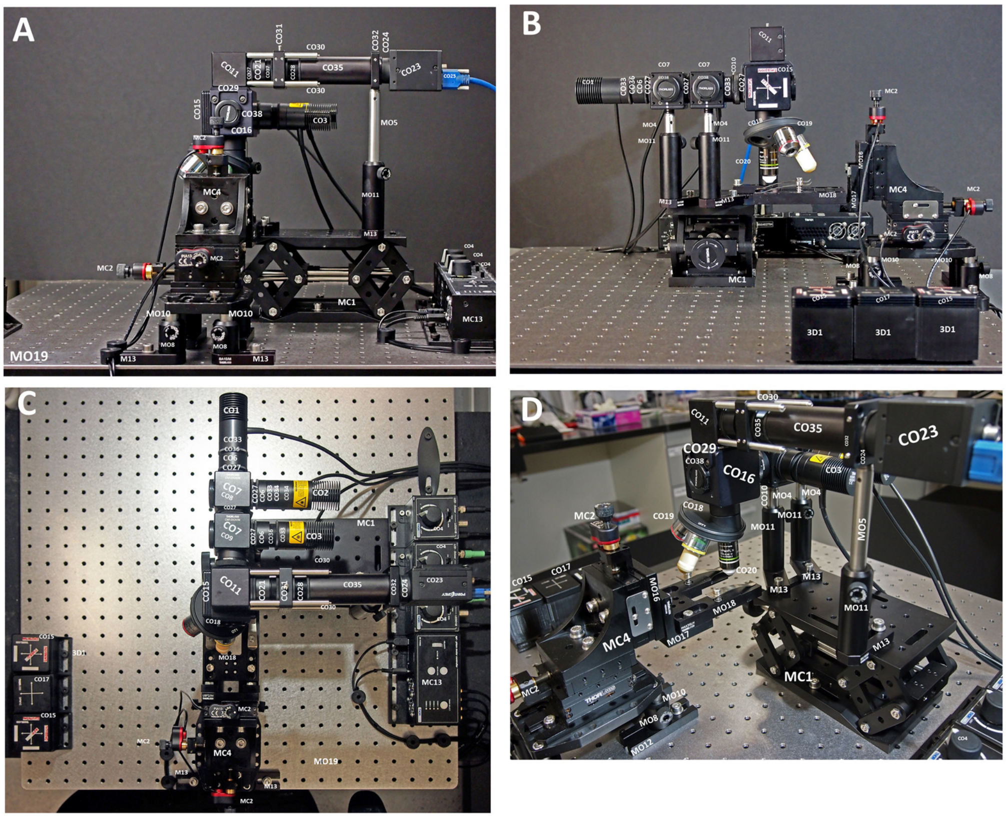

Figure 3. Mounting aspheric condenser lens for LED collimation.

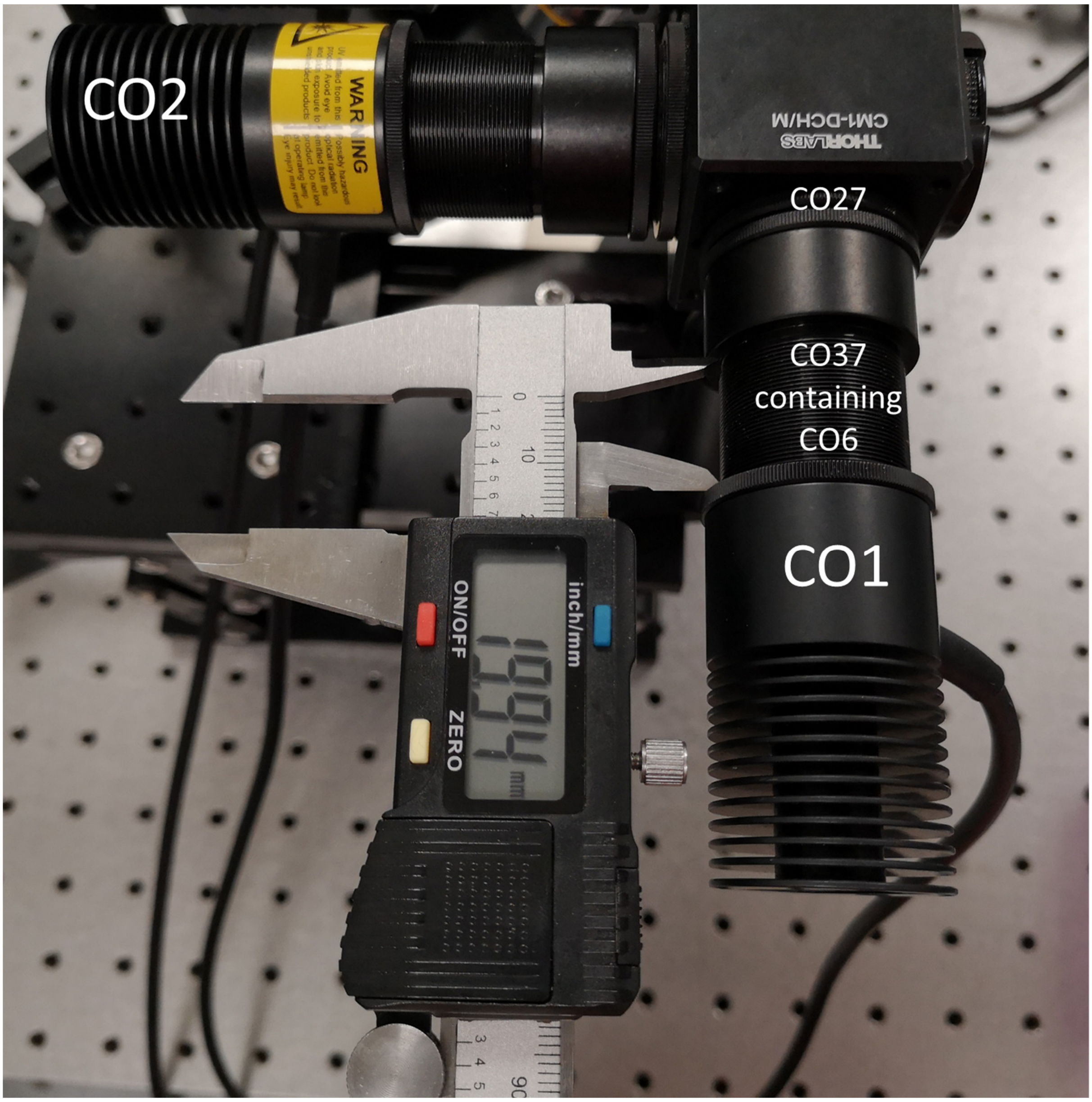

Figure 4. Testing the LED collimation

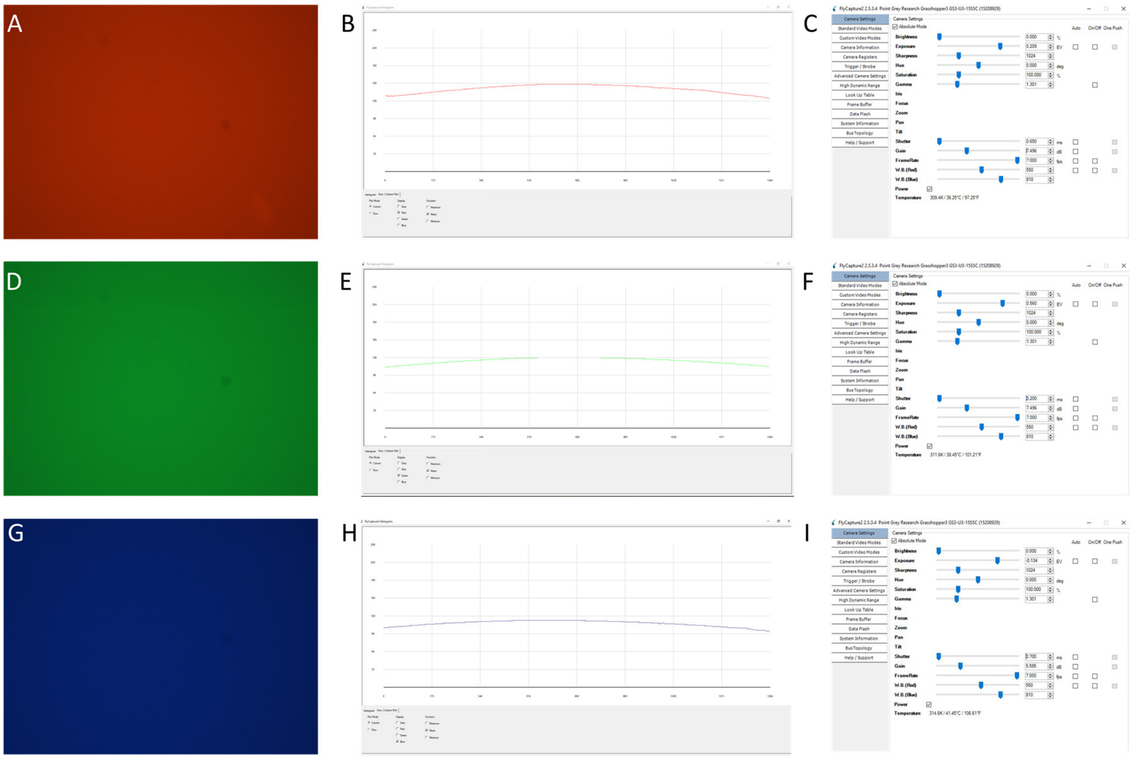

Figure 5. Determining the pixel size in ImageJ.

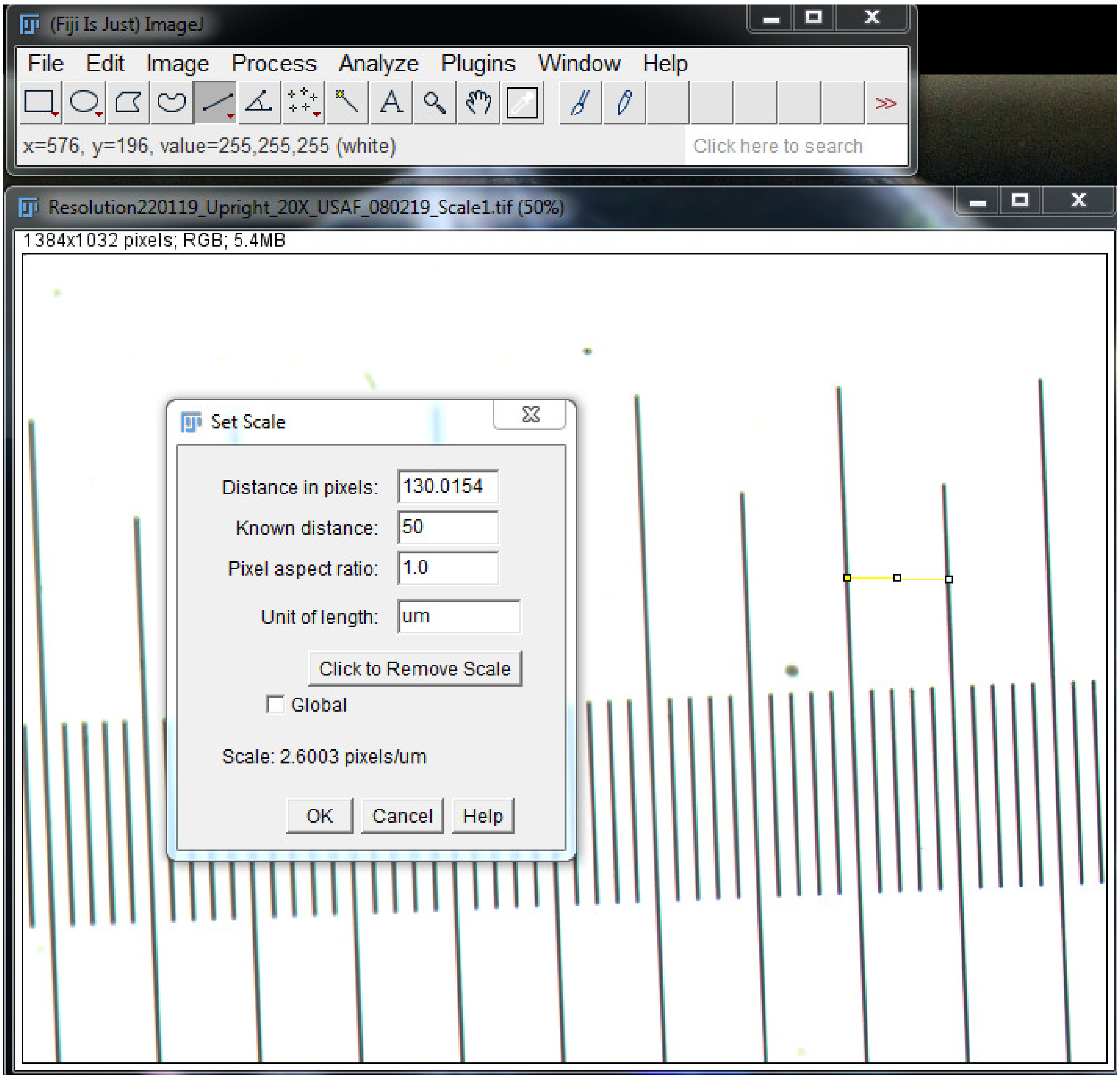

Supplement: Supplementary file 1: assembly and code [file rsos191949supp4.zip › SupplementaryFilesAssemblyAndCode/Flexiscope Assembly/Assembly instructions/Flexiscope_AssemblyInstructions.pdf]
